# 

**Published:** 1876-01

**Authors:** 


Annual Report of Surgeon General U. S. A. Pamphlet, pp. 16.

---

On Pigmentary Deposits in the Brain, resulting from Malarial Poisoning. By Wm. A. Hammond, M.D. Pamphlet, pp. 15.

---

On the Cause of Vice-President Wilson's Death. By William A. Hammond, M.D. Pamphlet, pp. 14.

